# *Helicobacter pylori* in the post-antibiotics era: from virulence factors to new drug targets and therapeutic agents

**DOI:** 10.1007/s00203-023-03639-0

**Published:** 2023-08-07

**Authors:** Omnia Momtaz Al-Fakhrany, Engy Elekhnawy

**Affiliations:** Pharmaceutical Microbiology Department, Faculty of Pharmacy, Tanta University, Tanta, 31527 Egypt

**Keywords:** Virulence, Urease, Neutrophil-activating protein (NAP), Outer membrane vesicles, Phytomedicine, Probiotics

## Abstract

*Helicobacter pylori* is considered one of the most prevalent human pathogenic microbes globally. It is the main cause of a number of gastrointestinal ailments, including peptic and duodenal ulcers, and gastric tumors with high mortality rates. Thus, eradication of *H. pylori* is necessary to prevent gastric cancer. Still, the rise in antibiotic resistance is the most important challenge for eradication strategies. Better consideration of *H. pylori* virulence factors, pathogenesis, and resistance is required for better eradication rates and, thus, prevention of gastrointestinal malignancy. This article is aimed to show the role of virulence factors of* H. pylori*. Some are involved in its survival in the harsh environment of the human gastric lumen, and others are related to pathogenesis and the infection process. Furthermore, this work has highlighted the recent advancement in *H. pylori* treatment, as well as antibiotic resistance as a main challenge in *H. pylori* eradication. Also, we tried to provide an updated summary of the evolving *H. pylori* control strategies and the potential alternative drugs to fight this lethal resistant pathogen. Recent studies have focused on evaluating the efficacy of alternative regimens (such as sequential, hybrid, concomitant treatment, vonoprazan (VPZ)-based triple therapy, high-dose PPI-amoxicillin dual therapy, probiotics augmented triple therapy, or in combination with BQT) in the effective eradication of *H. pylori*. Thus, innovating new anti-*H. pylori* drugs and establishing *H. pylori* databanks are upcoming necessities in the near future.

## Introduction

*Helicobacter pylori* (*H. pylori*) is a microaerophilic flagellated Gram-negative bacterium with a spiral shape which can switch from spiral to coccoid form. It inhabits the gastric lumen of about half of the human population globally. The helical shape of *H. pylori* is thought to enable it to survive in the human gastrointestinal tract. Also, the helix shape of *H. pylori* facilitates bacterial movement, while the coccoid form allows the colonization of the gastric epithelial cells, thus promoting its invasiveness. Moreover, *H. pylori* is able to form biofilms that lead to antimicrobial resistance, transmutations, and additional troubles with pathogen suppression (Baj et al. 2020; Abadi and Perez-Perez 2016).

Infection is expected to occur during childhood and may persist lifelong with the lack of proper antibiotic treatments (Šterbenc et al. 2019; Zhu et al. 2023). The predominance of *H. pylori* infections rises with age and varies extensively (10–90%) based on geographical regions being more remarkable in the developing populations (up to 88%) than in the developed ones (Hu et al. 2020). This could be attributable to variations in socioeconomic levels and lifestyles (Hooi et al. 2017). Various virulence factors have enabled *H. pylori* to adapt to the harsh environment in the human gut. These factors allowed the persistence of this bacterium in acidic environments, the permanent colonization of gastric mucosal surfaces, and enabled sustaining persistent *H. pylori* infections. Moreover, *H. pylori* has a number of virulence genes which express effector proteins that directly destroy the gastric epithelium (Whitmire and Merrell 2019). This will result in tissue damage and prolonged inflammation of the gastric mucosa that may sooner or later cause peptic ulcers and gastric carcinoma (Mehrotra et al. 2021).

Infections with *H. pylori* aren’t often accompanied by symptoms or warning signs. Though chronic infection may cause gastritis, and peptic and duodenal ulcers, and may even progress to mucosa-associated lymphoid tissue (MALT) lymphoma and also gastrointestinal carcinoma (Mehrotra et al. 2021). Being the highest risk factor for gastric carcinoma, the International Agency for Research on Cancer has categorized *H. pylori* as a class I carcinogen. Based on the Globocan 2018 records (Khazaei et al. 2020), gastric cancer is the 5th highest predominant cancer and the 3rd in mortality ratio. This highpoints the extreme necessity to get rid of this pathogen to prevent stomach cancers (Lee et al. 2016).

Additionally, *H. pylori* infections are associated with extra gastric ailments, including hematological, ocular, cardiovascular, neurologic, dermatologic, and liver diseases (Muhammad et al. 2017). Furthermore, recurrence is a severe problem opposing the effective elimination of *H. pylori* infections. The annual report of reinfection is about 8.7% and varies depending on age, geographical area, educational levels, percentage of infected household members, and the socioeconomic level of affected persons (Hu et al. 2020).

Predominantly, *H. pylori* eradication is desirable to lessen gastritis and inhibits its progression to gastric cancer and other extra gastric diseases (Malfertheiner et al. 2017).* H. pylori* infections could be treated with antibiotics. Even with antibiotic regimens, eradicating *H. pylori* remains a significant challenge (Hathroubi et al. 2020a; b). Conventional regimens involved two or three broad-spectrum antibiotics and a proton pump inhibitor (PPI), for example, omeprazole. While the standard triple therapy (clarithromycin, amoxicillin, or metronidazole and a PPI) has been recommended for years, currently, it isn’t effective in eradicating *H. pylori* infections. Still, the growing antimicrobial resistance has significantly reduced the efficacy of most of these antimicrobials and caused a considerable decline in eradication rates of *H. pylori* contagions. The latest studies reported alarming resistance levels to clarithromycin, metronidazole, and levofloxacin (Salillas and Sancho 2020; Abadi and Yamaoka 2018; Suzuki et al. 2019). In 2017, the WHO incorporated the clarithromycin-resistant *H. pylori* in the highest priority pathogens list. Furthermore, *H. pylori* display high genetic diversity, which has resulted from the extraordinarily high homologous recombination and mutation rates (Vital et al. 2022).

At the present time, the foremost challenge to eradicating *H. pylori* infections is antibiotic resistance, which impacts the efficiency of eradication treatments. This work will discuss *H. pylori* pathogenesis and virulence factors, the recently used and recommended antibiotic regimens, challenges to *H. pylori* eradication, how to face antibiotic resistance and summarize the latest advances in *H. pylori* management*.*

### Pathogenesis and virulence of *H. pylori* infections

*H. pylori* has a number of virulence factors that enable its survival in the stomach, including urease, and factors that enable its motility and adherence to the gastric epithelium, such as flagella and adhesins. Such factors generally permit amendment to the aggressive conditions of the stomach and maintain persistent infections. Moreover, *H. pylori* have various virulence genes that are expressed into effector proteins, which impede the gastric epithelium. Some factors play a significant role in *H. pylori* pathogenicity (Vital et al. 2022). Thus, the pathogenesis of *H. pylori* infection and disease outcomes is mediated by a multifaceted interaction between host, environmental and bacterial virulence. Herein, we will discuss some of the significant* H. pylori* virulence factors and their role in microbial pathogenesis.

### Gastric epithelial cells: the first‑line defense hurdle

**Fig. 1:**
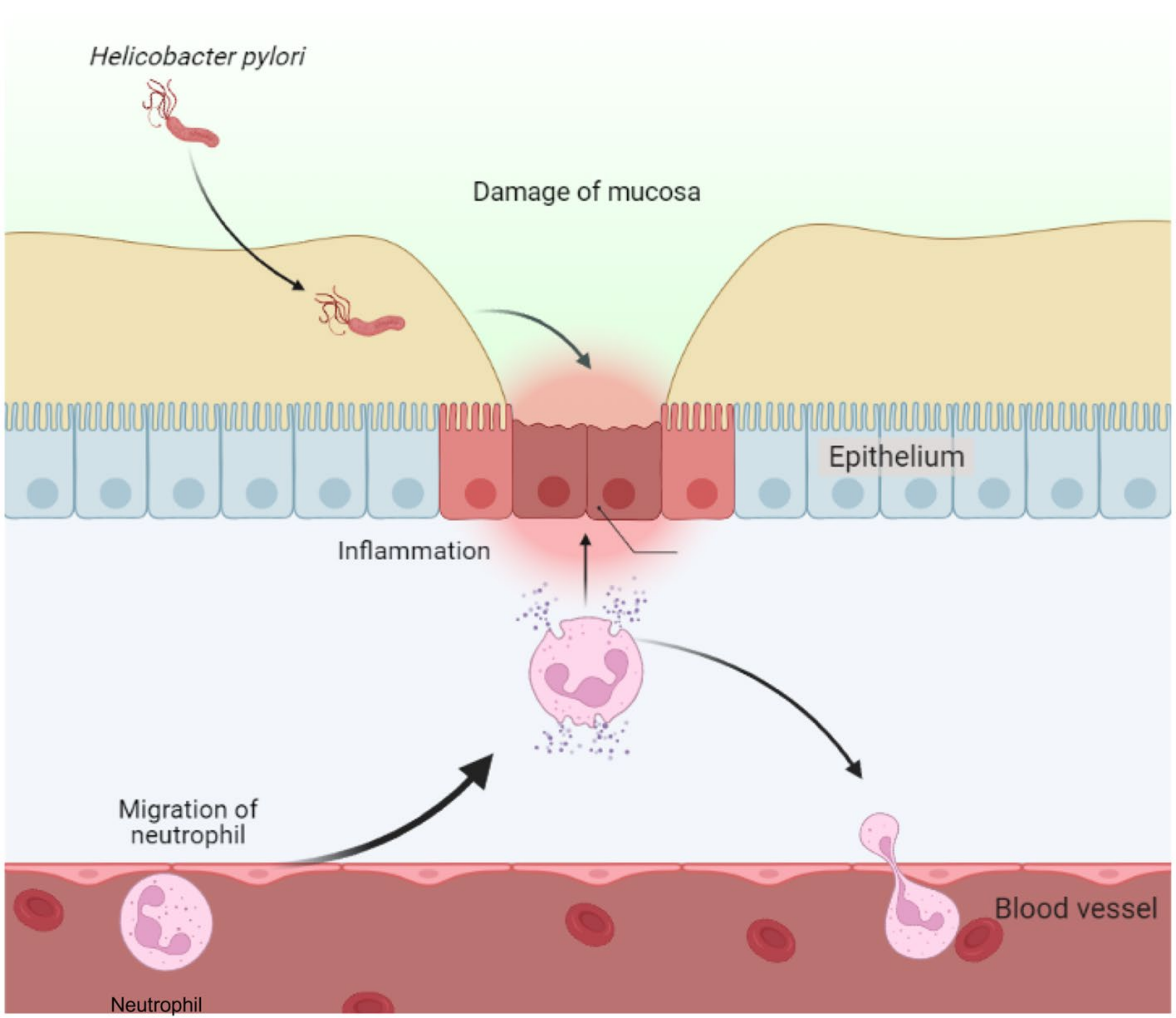
Pathogenesis of *H. pylori*

The human gastric epithelial cells act as a barrier that inhibits the movements, adhesion, and proliferation of the invading pathogens thru its tight structure**.** Pathogenic microbes such as *H. pylori* can disturb this barrier by releasing soluble detrimental constituents. Also, it can adhere to various receptors in the epithelial cells and induces many signaling pathways. Infection by *H. pylori* relies on (1) adaptation to the gastric acidity, (2) motility and invasion of the gastric epithelial barrier, (3) binding to particular receptors, (4) tissue destruction and other harmful health impacts (Fig. 1). Consequently, to cause infection, *H. pylori* must stay viable in acidic conditions of the gastric mucosa, adhere to gastric epithelial cells, and produce detrimental toxins/effector proteins (Sharndama and Mba 2022).

*H. pylori* releases several effector proteins/toxins that include outer inflammatory protein (OipA), neutrophil-activating protein A (NepA), blood group antigen-binding adhesion (BabA), vacuolating cytotoxins (VacA), sialic acid-binding adhesins (SabA), a cytotoxin-associated gene product (CagA), outer membrane vesicles (OMV), outer membrane protein (OMP), and high-temperature requirement A (HtrA) (Xu et al. 2020). Other *H. pylori* OMPs include *Helicobacter* OMPQ (HopQ), *Helicobacter* OMPZ (HopZ) and the *H. pylori* outer membrane (Hom) family proteins (HomA), HomB, HomC, and HomD. Xu et al. (2020) demonstrated the ability of HomB to stimulate the release of IL-8.

Remarkably, attachment, colonization of *H. pylori* in the stomach, and also the formation of biofilms are controlled by adherence-associated lipoprotein A and B (AlpA/AlpB). Besides, other adhesins are involved in the virulence of *H. pylori*. Recently, Baj et al. (2020) revealed that LacdiNAc-specific adhesin (LabA) enables *H. pylori* to adhere to the gastric epithelial cells. Moreover, Kinoshita-Daitoku et al. (2021) showed that *H. pylori* persistence in the stomach microenvironment is affected by Hpne4160, non-coding RNA. This study revealed that elevated expression of OMPs and *cagA* in chronic *H. pylori* infection was attributable to the declined expression of this adhesin.

### Colonization

A critical step in establishing *H. pylori* contagion is adherence to the gastric epithelium. Fundamentally, *H. pylori* outer membrane adhesins interact with gastric mucins and the gastric mucus membranes’ receptors. *H. pylori* adhesins include *H. pylori* OMP, blood group antigen-binding adhesin (BabA), and other proteins. These adhesins interact with the host epithelial cells' receptors. This attachment step protects the invading pathogen from clearance mechanisms, such as gastric peristalsis, constant detaching, and replacement of the mucus layer and the bulk liquid flow. Moreover, it provides nutritional sources to the invading pathogen and enhances the entry of toxins and other effector proteins (Ansari and Yamaoka 2020). Above and beyond, *H. pylori* employs its flagella in motility and adhesion mechanisms. Flagella facilitate the movement of invading pathogens to the mucus membranes, where these secrete adhesins to enable colonization of the gastric epithelium (Huang et al. 2016). Accordingly, it is considered an important virulence factor related to pathogenicity, especially in the early steps of *H. pylori* invasion. Interestingly, it has been revealed that non-flagellated *H. pylori* mutant strains are unable to colonize the gastric epithelial cells giving evidence that bacterial movement is crucial for further pathogenicity and infectivity (Saxena et al. 2020).

### Survival in gastric acidic environments

*H. pylori* flagellum is a fundamental factor allowing bacterial movement and chemotaxis. *H. pylori* has a package of 2–6 unipolar sheathed flagella. The length of each flagellum is just about three μm, and the sheath protects the bacteria from gastric acidity (Baj et al. 2020). In the relatively high percentage of acid in the stomach, *H. pylori* uses its flagella to protect itself from the gastric acidic environment (Saxena et al. 2020). In acidic pH, the flagella swim faster due to the proton motive force that drives their proteins motor. It has been reported that the exact flagella number affects the speed of bacterial cells’ motility (Martínez et al. 2016). Any possible mutations in the genes encoding the production of flagella, such as *fliD*, *flaA*, and *flaB*, may disrupt the colonization capacity of the invading pathogen. For instance, the *flaA* mutant strain of *H. pylori* can't have any flagella, while *flaB* lacking strains could produce flagella (Brito et al. 2019). However, a decline in the movement, adhesion, and colonization ability of *flaB* lacking strains was revealed. Along with motility, *H. pylori* flagella have a crucial role in biofilm formation and induction of inflammatory responses and immune evasion (Gu 2017; Hathroubi et al. 2020a). Quite a lot of flagellins, which constitute bacterial flagella, including HpA, FlaA, or FlaB, may stimulate humoral immune responses and induce specific antibody secretion. *H. pylori* strains which are highly motile, were shown to stimulate an elevated production of IL-8. Furthermore, genes encoding flagellar production might regulate other virulence factors, for instance, adhesins (Baj et al. 2020).

### Shape switching

Conversion of *H. pylori* from the helical shape to the coccoid shape occurs in antagonistic conditions. Several studies reported that *H. pylori* could survive in adverse conditions by switching its shape (Pinto et al. 2015). The spherical form is generally non-cultivable, replicable, antibiotic-resistant, and persistent for a sustained period resulting in severe hurt to the stomach (Krzyżek and Grande 2020). On the other hand, the helical form of *H. pylori* is cultivable. Thus, the helical cultivable *H. pylori* is usually identified by direct electron microscopy and several molecular procedures (Krzyżek and Grande 2020).

### Outer membrane vesicles (OMVs)

Outer membrane vesicles (OMVs) are small, circular structures present on the cell surface of *H. pylori* and various Gram-negative bacteria. OMV consists of lipids, proteins, toxins, and outer membrane proteins (OMPs). Occasionally, OMVs may have extracellular DNA (eDNA). Usually, OMVs are formed in the course of stress responses. OMVs have a crucial role in cell proliferation, viability, and the release of cytokine IL-8. Also, OMVs were demonstrated to promote bacterial survival, bacterial antibiotic resistance, DNA transmission, and stimulation of apoptosis (Chmiela et al. 2018). Recently, Murray et al. (Murray et al. 2020) reported the protective role of *H. pylori* OMV against some toxic substances, e.g., hydrogen peroxide and the bactericidal antimicrobial peptide LL-37 produced by epithelial cells. Furthermore, *H. pylori* OMV defends against both levofloxacin and clarithromycin in a dose-dependent way (Sharndama and Mba 2022). In general, OMV proteins are categorized into five classes based on the genes encoding these proteins. The first class includes the outer membrane porins (Hop), Hop-related proteins (Hor), the second class includes Hof, the third class comprises the *H. pylori* outer membrane proteins (Hom), the fourth class contains iron-regulated OMPs, and the fifth class is made up of the efflux pump OMPs (Oleastro and Ménard 2013).

### Outer membrane protein (OMP)

Outer membrane proteins (OMPs) are involved in *H. pylori* virulence, proposing their possible role as vaccine or drug targets. Several OMPs have been reported to take part in the virulence of *H. pylori* (Vital et al. 2022). OMPs are crucial in bacterial adhesion to the host gastric epithelium and promote cag pathogenicity island induction of the inflammatory immune responses and prompt signaling in affected host cells (Imkamp et al. 2019). *H. pylori* have approximately 64 OMPs that are assembled into five gene families. The OMP Family 1 consists of the *hop* and *hor* genes that encode adhesins, including the BabA/B/C, SabA/B, and AlpA/B. Expression of OMPs is controlled by genetic recombination or strand mispairing that occurs in the dinucleotide repeat regions of their respective encoding gene (Marcus et al. 2018). Family two genes code for OMPs of unidentified role. Nevertheless, the OMP family five genes encode the efflux pump proteins. Accordingly, they contribute to antibiotic resistance (Bina et al. 2000).

### Outer membrane porins

Outer membrane porins have a crucial role in adhesion to host cells. The family of outer membrane porin (HOP) adhesion proteins includes several adhesins that adhere to the specific receptors on their host cell membranes. This class consists of the sialic acid-binding adhesin (SabA), blood group antigen-binding adhesin (BabA), HopZ, outer inflammatory proteins A (OipA), HopQ, and the adherence-associated lipoprotein A (AlpA) (Costa et al. 2015). SabA and BabA are widely investigated and have great importance. Both enable adhesion and colonization of the gastric epithelium (Sharndama and Mba 2022). BabA attaches to H, Lewis b (Leb) and ABO blood group antigens on RBCs, and gastric epithelial cells. This binding enables the transport of both VacA and CagA to the epithelial cell thru the T4SS system (Ishijima et al. 2011). SabA recognizes Sialyl-Lewis x and Sialyl-Lewis antigens found on gastric epithelial cells. Also, OipA stimulates the release of interleukin-8 (IL-8), inducing inflammation and preventing apoptosis (Al-Maleki et al. 2017). HopQ is another important outer membrane porins that play a vital role in the transport of virulence factors inserted by CagA into the host cells via the T4SS system. Hom stimulates adhesion and the release of IL-8 and further inflammatory mediators (Sharndama and Mba 2022).

### Urease

Urease is a significant virulence factor of *H. pylori* (*Ansari &Yamaoka, 2020)*. Urease has a central role in bacterial metabolism and colonization in the gastric mucus membranes. *H. pylori* produces two types of urease; one is found in the bacterial cytoplasm (internal urease), and the other is present on the bacterial surface (external urease) (Sharndama and Mba 2022). The external urease is principally secreted during the degradation of other bacterial cells and is active at pH (5.0–8.0). However, the internal urease is activated at pH (2.5–6.5) (Miller and Maier 2014). Urease mediates the breakdown of urea into ammonia (NH_3_) and carbamate. Then carbamate is broken into ammonia and carbonic acid and ultimately promotes the alkalization of the acidic gastric environment; the entire process is controlled by the availability of nickel ions. The full enzymatic action of one urease molecule needs 24 nickel ion cofactors (Brito et al. 2019).

Urease has a critical role in *H. pylori* colonization of the gastric mucosa. This is due to its ability to protect *H. pylori* from the acidic environment in the stomach because of enhanced ammonia production. Also, it supports bacterial nourishment thru the deliverance of the host metabolites. Still, urease activates the production of proton motive force throughout urea hydrolysis (Sharndama and Mba 2022). Moreover, urease acts as a host immunomodulator by various mechanisms, for example, stimulating chemotaxis of neutrophils and monocytes, altered opsonization, apoptosis, platelet activation and enhanced secretion of cytokines (Wassermann et al. 2010). Urease activity in *H. pylori* is controlled by the pH of the surrounding gastric lumen and the accessibility of the nickel cofactor. Bacterial cultures enriched with nickel exhibit considerably enhanced urease activities (Belzer et al. 2005). Interestingly, it was reported that transforming *H. pylori* from its spiral to the coccoid form is an essential step for urease function and activity. *H. pylori* in the spiral form shows more significant activity compared with the coccoid form. However, more significant urease activity might induce pathological alterations within the gastric mucosa and tumors (Ghalehnoei et al. 2016).

### Vacuolating cytotoxins (VacA)

Vacuolating cytotoxin A (VacA) is a toxin that forms pores in host cells, depending on the exposure time. It enhances bacterial colonization and prolonged existence in the stomach (Baj et al. 2020). Acute exposure to VacA induces the autophagy pathway. However, chronic exposure stimulates the formation of impaired autophagosomes. It enhances the development of intracellular vacuoles, which support the prolonged existence of *H. pylori* in the prolonged persistence of bacterial infection. Vacuolation induced by VacA protein can induce several pathogenic effects on the host gastric epithelial cells, including vacuolation cytotoxicity, necrosis, and apoptosis (McClain et al. 2017). Besides, it causes disruption of endocytic trafficking, efflux of several ions, disruption of normal mitochondrial functions, and depolarization of the plasma membrane potential (chloride, bicarbonate, and urea) (Foegeding et al. 2016). Furthermore, vacuolation of the cell cytoplasmic membranes makes the cells exposed and liable to urease activities. Additionally, VacA considerably affects the immune system by impeding stimulation and proliferation of T cells and B cells and provoking apoptosis of macrophages thru inhibiting the IFN-γ signaling. As well, VacA stimulates the excessive release of IL-8 (Schauer et al. 2010). Moreover, VacA enhances the differentiation of regulatory T cells into effector T cells, which causes the persistence of *H. pylori* infections. Moreover, the gastric epithelium is attacked by VacA-containing endosomes that consequently enhance various degradative pathways to induce host inflammatory and immunological reactions and destroy different host organelles (Vital et al. 2022).

### Cytotoxin-associated gene A product (CagA)

One more significant virulence factor of *H. pylori* is the cytotoxin-associated gene product (CagA). Adhesion of *H. pylori* to the epithelial cells of the stomach stimulates cagA expression that is usually associated with high inflammatory responses, damage of gastric mucosal cells, and gastrointestinal injuries provoked by *H. pylori* (Raghwan and Chowdhury 2014). The oncoprotein (CagA), together with a type four secretion system (T4SS) has a crucial role in tumorigenesis. CagA is encoded on the *cag* pathogenicity island (*cag*PAI). CagA is inserted into the host cell by pili made by the T4SS (Baj et al. 2020). In the cell, it triggers cellular modifications which change the arrangement of the whole cytoskeleton, and impair cellular motility, proliferation, and apoptosis. Also, the oncoprotein CagA can modify the tumor suppressor pathways in gastric epithelial cells. CagA prompts carcinogenesis disturbing the activities of tumor suppressor proteins, for example, the run-related transcription factor 3 (RUNX3) and the apoptosis-stimulating protein of p53 2 (ASPP2). Furthermore, Choi et al. (2019) demonstrated that increased gastric cancers might be related to increased expression of the transcription factor CDX1, which is prompted by the CagA positive strains. *H. pylori* strains are frequently grouped into *cagA*-positive strains and *cagA*-negative strains. *cagA* is often related to mucosal inflammation and serious gastrointestinal clinical outcomes, including; peptic ulcers and gastric cancer (Sharndama and Mba 2022). Also, *cagA*-positive strains have higher motility than *the cagA*-negative ones, which indicates its association with bacterial motility. Overall, CagA has cytotoxic and immunomodulatory actions (Vital et al. 2022; Sharndama and Mba 2022).

### Catalase (KatA)

Catalase (KatA) is an abundant enzyme in plant and animal cells. KatA acts by converting hydrogen peroxide (H_2_O_2_) into water (H_2_O) and molecular oxygen (O_2_). In general, catalase is a highly expressed protein* by H. pylori* isolated from the human gastric mucosa. Lekmeechai et al. (Lekmeechai et al. 1837) showed that KatA triggered the neutralization of H_2_O_2_ and NaCl, thus protecting *H. pylori* from oxidative stresses and activities. Also, it protects *H. pylori* from complement-mediated damage, enabling bacterial longevity and colonization (Richter et al. 2016). Besides, catalase plays a significant role in several pathological processes, including; inflammation, apoptosis hindrance, along with tumor formation via induction of mutagenesis. Catalase also enhances the persistence of *H. pylori* in the macrophage phagosomes. Likewise, *H. pylori* catalase protects the bacteria from harmful long-chain fatty acids and their toxic metabolites. Over and above, it protects *H. pylori* from phagocytosis; and has a critical immunomodulatory role. Its activity is regulated by Fur protein and iron levels. Previous studies revealed that Fur-deficient strains of *H. pylori* growing on a low iron culture media showed significantly reduced catalase activity. Catalase can present its activities in the cytoplasm, periplasm; and also on the surface of the cell (Baj et al. 2020).

### Neutrophil-activating protein (NAP)

Neutrophil-activating protein (NAP) has a crucial role in the virulence of *H. pylori*. NAP has bacterial protective and host inflammatory activities. NAP activated several inflammatory reactions via stimulation of various immune cells, including neutrophils, monocytes, and mast cells, to prompt their oxidative and inflammatory activities. It prompts adherence of neutrophils to endothelial cells and also promotes dose-dependent production of ROS by neutrophils and monocytes (Baj et al. 2020). The oxidative stress prompted by NAP harms the gastric epithelium and destroys the invading *H. pylori* as well. Remarkably, it has been indicated that ROS can induce biofilm formation by *H. pylori* to protect itself from oxidative stress-induced toxicity. Thus NAP is recognized as a biofilm-related protein in *H. pylori.* It is involved in stimulated *H. pylori* biofilm production during oxidative stress to support antibiotic resistance. Hence, HP-NAP stimulates oxidative stress by activating of ROS production. Also, it protects the bacteria against impairments by oxidative stress thru several pathways (Zhao et al. 2021).

Interestingly, NAP displays structural similarities to ferritin and belongs to the DNA-protecting protein under severe conditions (Dps) family. NAP shows ferroxidase activities devoid of direct attachment, and the entire process produces hydroxyl radicals which have a protective role of *H. pylori* DNA from any destruction. Thus, NAP supports the survival of *H. pylori* by its role in DNA protection (Ceci et al. 2007).

Furthermore, NAP stimulates the degranulation of neutrophils. NAP triggers the secretion of various chemokines by neutrophils, including IL-8, macrophage inflammatory proteins (MIP)-1α and MIP-1β. Then, IL-8 further recruits immune cells, including neutrophils, to the infection site. Additionally, NAP stimulates monocytes' secretion of pro-inflammatory cytokines, including tumor necrosis factor-α (TNF-α), IL-12, IL-6, IL-8, and IL-23. Also, it stimulates mast cells to secrete IL-6 and histamine. The secretion of these factors considerably enhances the production of gastrin and pepsinogen and the consequent injury of the gastric mucosa. (Fu and Lai 2023). Besides, NAP induces mononuclear cells to produce tissue factor (TF) and plasminogen activator inhibitor-2 (PAI-2), resulting in imbalances between the coagulation cascade and fibrinolysis. These alterations might have a role in the progression of chronic gastritis induced by *H. pylori* infection thru disruption of fibrin removal and further impairment of the healing processes. The capability of NAP to stimulate innate inflammatory reactions indicates the pathogenicity of this protein during *H. pylori* infections thru inducing inflammation of gastric mucosa. In addition to induction of the innate immune responses, HP-NAP also induces adaptive immune responses. During *H. pylori* infection, NAP induces adaptive immune reactions through induction of T-helper type 1 (Th1) and cytotoxic T lymphocyte (CTL) activities promoting gastritis and tissue injury (Fu and Lai 2023).

### Other important virulence factors

There is a number of additional virulence factors which are involved in the pathogenesis of *H. pylori*, some of which are discussed below:

### Superoxidase dismutase (SOD)

Superoxide dismutase (SOD) is a virulence enzyme which protects *H. pylori* cells from reactive oxygen species (ROS). SOD enables the dismutation of superoxide to oxygen, avoiding excess toxic superoxide free radicals and supporting the maintenance of homeostasis. In contrast to catalase, *H. pylori* SOD is found only on cell surface. *H. pylori* SOD needs iron (Fe) as a cofactor for effective functioning. Any changes in the intracellular iron concentrations affect the SOD activities, making the cell susceptible to oxidative damage (Sharndama and Mba 2022). Furthermore, *H. pylori* SOD prevents cytokines release (thru down-regulation of the nuclear factor kappa B (NF-kB) activation pathway), along with macrophage inflammatory protein 2 (MIP-2), through stimulating macrophage activation (Stent et al. 2018). Besides, SOD expression is essential for survival, colonization, and growth in high oxidative stress. What’s more, there is an association between SOD activity and the start and progression of gastric ailments (Sharndama and Mba 2022).

### Phospholipases

Outer membrane phospholipase A of *H. pylori* has a significant role in the degradation of several lipids, causing bacterial permeability. *H. pylori* produces phospholipaseA1 (PLA1), phospholipase A2 (PLA2), phospholipase C (PLC) and phospholipase D (PLD), which destroy phosphatidylcholine in addition to phosphatidylethanolamine. This damages the mucus layer and results in progressive impairment of the gastric epithelial cells. Also, phospholipases trigger chronic inflammation, which further induces peptic ulcers. This enhances the colonization and survival of the invading bacteria. Interestingly, *H. pylori* strains which produce higher quantities of phospholipases, considerably raise the risk of long-term gastritis and consequent gastric malignancies (Lusini et al. 2005). Besides, *H. pylori* PLD triggers the ERK1/2 signaling pathway in the gastric epithelium, provoking cell damage (Sharndama and Mba 2022).

### γ-Glutamyl-transpeptidase (GGT)

Γ-glutamyl-transpeptidase (GGT) is another enzyme produced by *H. pylori* to promote the hydrolysis of glutamine to glutamate and ammonia and glutathione to glutamate and cysteinyl glycine. Glutamine breakdown enables the vacuolation process induced by VacA (Ling et al. 2015). GGT stimulates the production of ROS (mostly H_2_O_2_), prevents cell proliferation, and induces apoptosis as well as necrosis of the gastric epithelium through the unwarranted release of inflammatory mediators, including; cyclooxygenase-2 (COX-2), inducible iNOS, IL-8, IL-10, plus the epidermal growth factor-related peptides, causing cell cycle arrest in the G_1_ phase. Furthermore, GGT enables colonization and survival in the stomach thru modifying immune tolerance via inhibition of T cells and dendritic cell toleration. GGT inhibits CD4+ T cell proliferation, facilitates CD8+ T cells infiltration into the gastric milieu, and inhibits dendritic cells differentiation, activating modifications in their phenotype into the tolerogenic phenotype (Sharndama and Mba 2022).

### Cholesteryl α-glucosyltransferase (αCgT)

Cholesteryl α-glucosyltransferase (αCgT) is an *H. pylori* enzyme that plays a crucial role in the production of cholesteryl α-glucoside (αCGL) thru the addition of α-glucosyl to the host plasma membranes cholesterol. *H. pylori* can't produce cholesterol by itself. Instead, it extracts cholesterol from the host cell membranes. Then, αCgT activates the glycosylation of cholesterol, yielding cholesteryl glucosides (CGs). It is secreted by *H. pylori* as an inactive form that is activated by attaching to their host cell membranes. Glucosylation induced by αCgT shields *H. pylori* from phagocytosis and immunological attack. Furthermore, CGs trigger the release of inflammatory mediators. Also, CGs regulate the CD4+ cells, IL-4 and IFN-γ responses. Moreover, αCgT supports bacterial growth and persistence in the host by disturbing autophagosome–lysosome fusion (Lai et al. 2018). Simultaneously, the presence of GCs plays a crucial role in antibiotic resistance. Hence, αCgT could be a potential drug target for the treatment and eradication of *H. pylori* infections (Sharndama and Mba 2022).

### Heat shock proteins (Hsps)

Heat shock proteins (Hsps) are a set of proteins which act as molecular chaperones that support maintaining the appropriate structural and functional activities of the cell effector proteins. Also, Hsps controls apoptosis, autophagy, inflammatory responses, and carcinogenesis. Furthermore, Hsps keep bacterial cells from reactive oxygen species (ROS) and oxidative stress (Hsu et al. 2018). *H. pylori* have three main Hsps; HspB (Hsp60), HspA (Hsp10), and Hsp70. HspA plays an essential role in activating the urease enzyme produced by *H. pylori* during the infection process. Moreover, HspA attaches to nickel ions and bismuth. Accordingly, it might be a prospective target for novel anti-ulcer remedies. Also, the C-terminal domain of HspA has been described to participate in nickel sequestration and detoxification. As nickel is critical for colonization, HspA could be a target for novel *H. pylori* therapeutics (Schauer et al. 2010). Besides, HspA60 prompts the production of IL-8, IL-6, IL-10, TNF-α, and COX-2 and subsequent induction of inflammation in the gastric mucosa. Furthermore, Hsp60 enhances *H. pylori* attachment and colonization of the gastric mucosa (Baj et al. 2020).

### Recently used and recommended antibiotic regimens against *H. pylori* and upcoming approaches

*H. pylori* infections are asymptomatic in many patients, but they can cause several gastrointestinal diseases, e.g., chronic gastritis, gastric adenocarcinoma, peptic and/or duodenal ulcers, and lymphoma. Therefore, physicians are challenged to select who should be tested or treated. Generally, treatment is indicated in the case of detection of *H. pylori* infections, even if the patients are asymptomatic (Pellicano et al. 2016). In industrial countries, the reinfection rate is 2% due to effective treatment. However, the global annual recurrence rate of *H. pylori* infections was more significant in the 2010s (4.8%) than in the 1990s (3.9%) and 2000s (4.4%). Therefore, the frequency of infected persons might be increasing. Moreover, *H. pylori* infection, if not eradicated, persists for a lifetime. Several consent reports have been issued in the preceding five years, mainly concentrating on managing *H. pylori* infections (Pohl et al. 2018). That’s why suitable treatment could have promising eradication rates without reinfection. Still, significant challenges facing the treatment and eradication of *H. pylori* infections include growing antibiotic resistance and compliance to recommended regimens, as shown in Table 1.

**Table 1:** The available *H. pylori* treatment strategies and promising upcoming approaches

Current drugs	Future therapeutic approaches
Standard triple therapy Sequential therapy	Using probiotics or PPI or medicinal plants with antibiotics
Levofloxacin triple therapy Bismuth quadruple therapy	Antibiofilm agents Novel targets

Several regimens are recommended for eradicating *H. pylori* infections. The standard conventional proton pump inhibitor (PPI)-based triple therapy (PPI and two antibiotics) is one of the most frequently used therapies (Hu et al. 2020). It consists of PPI, amoxicillin (AMX), and clarithromycin (CLR) or metronidazole (MTZ). However, PPI-based triple therapy offers low therapeutic efficacy. This regimen is suitable for regions with decreased CLR resistance (< 15% efficacy) and should continue for 10–14 days. In addition, the 2016 Maastricht V/Florence Consent Report and the 2016 Toronto Consensus (Fallone et al. 2016) recommended the bismuth-containing quadruple therapy (BQT) as the best choice first-line regimen for eradicating *H. pylori* in regions with higher or lower clarithromycin resistance due significant efficacy, tolerance, and safety (Hu et al. 2020). BQT regimen consists of fourteen-day bismuth quadruple therapy (PPI, bismuth, MTZ, and tetracycline (TC). Another acceptable regimen in case of high CLR resistance is using a broad-spectrum quinolone, levofloxacin (LVF) triple therapy (LVF, AMX, and PPI). Though, LVF triple therapy isn’t indicated as a first-line regimen due to the growing global resistance to LVF. Then again, in regions of declined LVF resistance and elevated CLR and MTZ resistance or in case of unavailability of bismuth, an LVF triple regimen is recommended (Fallone et al. 2019).

The 2016 Maastricht V/Florence Consent Report and the 2016 Toronto Consent (Fallone et al. 2016) recommended BQT or accompanying therapy (PPI, AMX, CLR, MTZ) if bismuth isn’t accessible as first-line therapy in case of 15%CLR. Also, sequential therapy is a promising treatment strategy that involves PPI and AMX for the first five days and PPI, MTZ, and CLR for the second period (Kamboj et al. 2017).

In case of failure of the first-line therapies, various second-line treatments can be applied. For instance, a bismuth quadruple therapy (PPI, bismuth, MTZ, and TC) is usually suggested when the standard triple therapy fails to give promising outcomes. A ten-day TC-LVF quadruple regimen is the ideal second-line regimen. It comprises esomeprazole (PPI) and bismuth salt, along with antibiotics (Lin and Hsu 2018). Moreover, the 2016 Maastricht V/Florence Consent Report recommends fluoroquinolone-AMX triple or quadruple therapy as second-line rescue therapy with comparable efficiency. It consists of LVF, AMX, PPI, and bismuth (just in quadruple therapy). Recently, the efficacy of other regimens, including; sequential, hybrid, concomitant therapy, high-dose PPI-amoxicillin dual regimen, vonoprazan (VPZ)-based triple regimen, probiotics supplemented triple therapy or combined with BQT, was evaluated.

### Challenges to *H. pylori* eradication

Recently, the *H. pylori* eradication rate of the standard treatment has been declining globally. Furthermore, the management of *H. pylori* has lots of challenges, involving antibiotic resistance, patient compliance, adverse effects, and recurrent infection. While antimicrobial resistance in *H. pylori* differs according to the geographic region, its rising prevalence has resulted in therapy failure and decreasing eradication rates (Tshibangu-Kabamba and Yamaoka 2021). Besides, to optimize *H. pylori* therapeutic regimens, the significance of gastric tumor relapse after eradication and susceptibility tests with molecular methods before treatment must be considered (Liang et al. 2022).

### Facing antibiotic resistance of *H. pylori*

*H. pylori's* resistance to antibiotics has overextended to frightening levels globally. Overuse of antibiotics, mutations, changes in the host microbiome and incomplete treatment have resulted in increased antibiotic resistance (Tehlan et al. 2020). Antibiotic resistance is the foremost challenge to current eradicating regimens (Vital et al. 2022). In 2017, the World Health Organization (WHO) considered *H. pylori* as one of the twenty pathogenic bacteria which represent a major hazard to human health owing to antibiotic resistance (Tacconelli et al. 2018). Recent studies reported alarming levels of primary and secondary resistance to CLR, MTZ, and lVF (exceeded 15%) in all the WHO regions, with resistance rates of > 15% to both CLR and MTZ in some regions (Savoldi et al. 2018). However, resistance rates to AMX, TC, and furazolidone stay declined. Elevated clarithromycin resistance has resulted in a declining eradication rate of the conventional first-line triple therapy (containing clarithromycin) to less than 70% (Liang et al. 2022). With the alarming emergent levels of antibiotic resistance and failure of the standard triple therapy regimen, there is an urgent need to find further alternative efficient drugs for fighting this deadly global pathogen. The development of the *H. pylori* vaccine is also a hot research issue (Xu et al. 2020).

### Susceptibility-guided therapy or tailored therapy

The efficiency of antimicrobial agents is governed by bacterial susceptibility to certain drugs, formulations, doses, treatment duration, adjuvant therapies, and recurrence rates. Antibiotic susceptibility testing is the best way to adjust antibiotic usage in *H. pylori* management. Susceptibility-guided treatment is thought to have great efficiency rates owing to its dependence on outcomes of antibiotic resistance and avoidance of antibiotic misuse. Besides, recent studies revealed the efficacy of susceptibility-guided therapy as the first- or second-line eradication regimen, particularly in areas with elevated antibiotic resistance (Hu et al. 2020). Moreover, *H. pylori* resistance surveillance programs should be established globally to deliver local resistance reports that might help prepare treatment guidelines in certain regions. Even so, antibiotic susceptibility tests may be a further challenge. As it generally involves biopsy, and also the cultivation of *H. pylori* is a costly and time-consuming process (Roszczenko-Jasińska et al. 2020). On the other hand, empirical therapies have little efficacy because of the varying antibiotic resistance profiles of *H. pylori* globally. Also, excessive antibiotic usage in *H. pylori* treatment has to be avoided. This is due to its impacts on the regimen efficacy and increasing the probability of new multidrug-resistant strains (Roszczenko-Jasińska et al. 2020).

Recently, dual priming oligonucleotide-based multiplex polymerase chain reaction (DPO-PCR) has been recommended. DPO-PCR can identify *H. pylori* infections in addition to clarithromycin resistance depending on the A2142G and A2143G mutations of 23S rRNA. In Korea, tailored therapy based on DPO-PCR displayed excellent efficacy and cost-effectiveness as a first-line therapy of *H. pylori*. Also, the DPO-PCR technique is associated with susceptibility tests based on cultures (Ong et al. 2019). Furthermore, GenoType HelicoDR is a molecular technique which identifies *H. pylori* infection in addition to CLR and LVF resistance. Recent research demonstrated that the higher eradication and the lower resistance rates of tailored therapy depend on the GenoType HelicoDR test compared with the empiric triple regimen (Delchier et al. 2020).

### Recent progress in treating *H. pylori* infections

#### Novel components in the standard *H. pylori* antibiotic regimens

A novel approach to eradicate *H. pylori* infection is dual therapy with amoxicillin and a PPI. The dual therapy has proven encouraging outcomes regarding *H. pylori* eradication and reducing the concentration of antibiotics used, thus minimizing the effects on the host microbiome (Suzuki et al. 2019). Formerly, this strategy did not have satisfactory results. The effective strengthening point for this dual therapy is the replacement of traditional PPIs with the novel PPI vonoprazan, which is a potassium-competitive acid blocker with powerful, long-lasting inhibitory effects on acid production and creates neutral gastric pH appropriate for the growth and survival of *H. pylori.* Neutral pH allows dormant *H. pylori* to go into the replication stage and become antibiotic-sensitive (Roszczenko-Jasinska et al. 2020) showed that a seven-day treatment with AMX-vonoprazan resulted in 93.8% eradication of *H. pylori* infections. Restrictions of this dual regimen may be inaccessible in addition to penicillin-allergic reactions (Suzuki et al. 2019; El-Banna et al. 2019).

Rifabutin, a broad-spectrum derivative of the antibacterial rifamycin, is usually used as an antituberculosis preparation against rifampicin-resistant Mycobacterium tuberculosis strains. Also, it is utilized against *M. avium* in HIV patients. Due to its high in-vitro efficacy against *H. pylori*, its stability in the gastric lumen, and rifabutin-resistant strains that have infrequently been isolated, the rifabutin-based regimen has been regarded as a possible choice to treat *H. pylori* infections. Rifabutin inhibits the ß-subunit of RNA polymerase encoded by the *rpo*B gene. So far, the triple rifabutin regimen has been a saving treatment after many failures (Pellicano et al. 2016). Graham et al. (2020), indicated that supplementing a dual regimen (AMX and PPI) with rifabutin considerably improved the effectiveness.

Additionally, adding bismuth to rifabutin-based triple therapy (AMX, PPI, rifabutin) significantly enhanced the efficacy. Therefore, rifabutin-based treatments have a perspective as a first- or second-line empiric therapy for *H. pylori*. Yet, some limitations concerning rifabutin include highly high cost, severe side effects (myelotoxicity, leucopenia), and the extensive use of rifabutin could result in resistant *H. pylori* strains (Roszczenko-Jasińska et al. 2020; Abdelaziz et al. 2019).

### Antimicrobial peptides (AMPs)

Recently, antimicrobial peptides (AMPs) have been confirmed as effective antibiotic alternatives for overcoming resistant microbes (Xu et al. 2020; Elekhnawy et al. 2021a). AMPs are small peptides secreted by cells of many living organisms that mediate innate immune responses. Also, AMPs possess broad-spectrum activity on several pathogenic microorganisms. Antimicrobial Peptide Database (APD) comprises 3324 AMPs (391 bacteriocins/peptides from bacteria, 364 from plants, 22 from fungi, five from archaea, eight from protists, and 2446 from animals, together with some synthetic peptides (Sukri et al. 2021).

AMPs have renewed our understanding of the relationship between immune defense mechanisms and human diseases. AMPs are small peptides that generally comprise less than 100 amino acids. AMPs have an amphipathic structure which enables cationic AMPs to interact specifically with the anionic bacterial cell membranes and enhance their permeability, causing cell death unaccompanied by affecting neutral eukaryotic cell membranes. In addition, certain AMPs inhibit various cellular processes, including transcription, translation, and cell wall synthesis (Jhaveri et al. 2015; Elekhnawy et al. 2021b).

In 2019, Neshani et al. (2019) reported the anti-*H. pylori* activities of 22 antimicrobial peptides, of which three peptides had the strongest action, namely; pexiganan, tilapia piscidin 4 (TP4), and PGLa-AM1. These are small cationic 5-kDa peptides with a high positive charge and isoelectric point. Although most AMPs are active on some *H. pylori* antibiotic-resistant strains, several drawbacks to their clinical use, including; their degradation by host and bacterial proteases equally. Some AMPs can produce cytotoxic effects in human cells. Additionally, after prolonged use, bacteria can resist AMPs (Pero et al. 2019). Even with disadvantages, AMPs have proven synergistic effects in combination with many antibiotics. Nevertheless, further in vivo studies are still required.

### Probiotics

Recent research has focused on the use of probiotics to eradicate *H. pylori*. Probiotics are defined as living microorganisms, when used in suitable quantities, that produce health benefits (Liang et al. 2022). Several studies have demonstrated the benefits of probiotics on human health, principally by improving the host gastrointestinal tract microbiota, the immune responses and inhibitory activities against the attacking pathogens. Recently, probiotic supplementation has emerged in *H. pylori* eradication regimens (Liang et al. 2022).

**Fig. 2:**
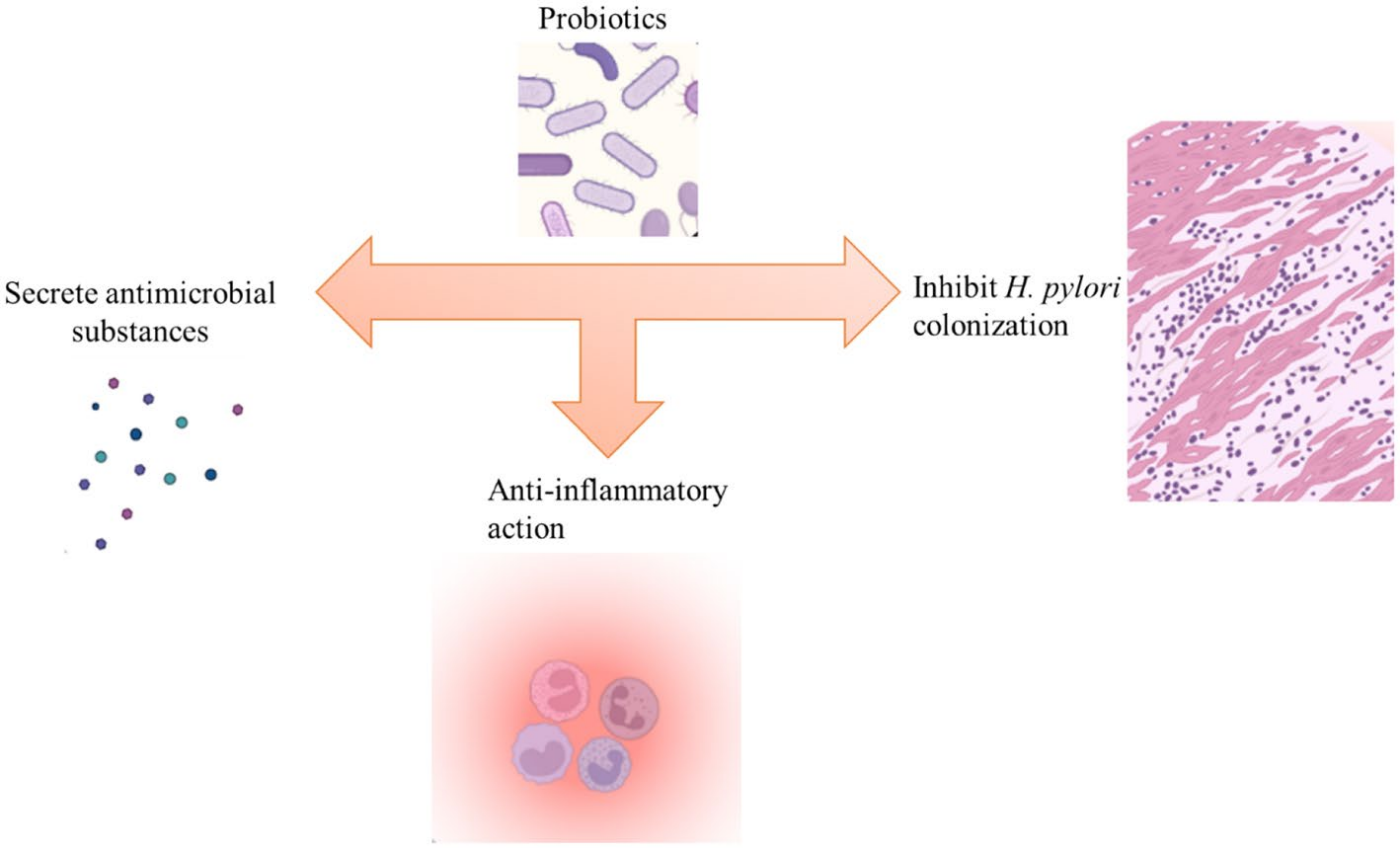
Representative illustration of the possible mechanisms of action of probiotics to eradicate *H. pylori*

The possible activities of probiotics to eradicate *H. pylori* are summarized in Fig. 2. Probiotics may augment the barrier effects of the gastric mucosa that stands as the first defense line facing invading pathogens. Various probiotics can enhance the tight junction protein expression, stimulate mucin and mucus secretion, and promote the barrier effects of the gastrointestinal mucosa (Suez et al. 2019). Also, probiotics, such as *Lactobacillus bulgaricus*, *Lactobacillus reuteri*, and *Lactococcus lactis*, can secrete peptide and non-peptide antimicrobial compounds that inhibit both the growth and the adherence of *H. pylori* to the gut mucosa. Such antimicrobials include hydrogen peroxide, lactic acid, short-chain fatty acids (SCFAs), and bacteriocins. The anti-*H.pylori* effects of lactic acid and SCFAs depend on the un-dissociated forms of such organic acids that inhibit *H. pylori* urease activity and cause cell damage (Liang et al. 2022).

Furthermore, probiotics can inhibit *H. pylori* adhesion and colonization through competition for the adhesion sites and the formation of co-polymers with *H. pylori* to facilitate its excretion (Liang et al. 2022). Moreover, some probiotics may alleviate the inflammation induced by *H. pylori* infection, improve the immune responses and reduce the adverse effects and consequently increase patient compliance. Sustained inflammation plays a key role in the pathogenesis of *H. pylori*. Probiotics prevent the expression of inflammatory cytokines and enhance histological inflammatory responses that continue after cessation of probiotic supplements (Liang et al. 2022). Besides, several studies report the ability of probiotics to hinder the expression of *H. pylori* virulence genes, such as *ure*B, *vac*A, and *sab*A (Roszczenko-Jasińska et al. 2020).

Various recent investigations have recommended the application of probiotics either alone or in conjunction with multiple antibiotics. Buckley et al. revealed that *L. reuteri* DSM 17648 significantly decreased the gut bacterial burden and relieved dyspeptic signs and symptoms. It was indicated that *L. reuteri,* in combination with a proton pump inhibitor, led to the eradication of *H. pylori* in 12.5% of patients. Although the use of probiotics only may have some activity against *H. pylori*, they can’t be used as antibiotic alternatives in the eradication regimen. However, probiotics supplementation to the eradication regimen could improve the effectiveness of antibiotic treatment (Zhang et al. 2015). In this regard, Zhou et al. (2019) explored the use *Saccharomyces boulardii* in conjunction with the triple therapy to eradicate *H. pylori*. The authors reported a substantial improvement in the eradication rate and a decline in the adverse effects compared with the standard triple therapy alone. Also, *Lactobacillus* can be used as adjuvant therapy to enhance the effectiveness of triple therapy and decrease the frequency of drug-induced diarrhea (Fang et al. 2019).

Furthermore, *H. pylori* eradication could result in multiple alterations in the intestinal microbiota. However, supplementation of the triple therapy with *Bacillus subtilis* and *Enterococcus faecium* possibly will restore the gastrointestinal microbiome (Hu et al. 2020). Still, there are many questions concerning probiotics, dosages, duration, efficacy of specific probiotic strains, and possible interactions between probiotic strains and antibiotics (Roszczenko-Jasińska et al. 2020).

### Phytomedicine

The application of natural products in *H. pylori* eradication is attaining a good reputation because of low side effects and toxicities. Traditional folk medication, particularly traditional Chinese medicine (TCM), is the leading light for employing medicinal herbs to fight several infections. Several studies have evaluated the effectiveness of many crude plant extracts in *H. pylori* treatment. The majority showed weak to moderate activities. However, the extract of *Impatiens balsamina* L., a Taiwanese folk medical herb, exhibited the highest activity (Zhang et al. 2015). Also, patented Chinese medicine containing *Chenopodium ambrosioides* L. (CAL) and *Adina pilulifera* (AP) is frequently utilized in *H. pylori*-induced gastritis and peptic ulcers.

Furthermore, Ye et al. (2015) have confirmed the antimicrobial activity of *C. ambrosioides* alone against *H. pylori*. Besides, Kouticheu Mabeku et al. (2017) reported the activity of *Bryophyllum pinnatum* against *H. pylori* due to its anti-ulcer activities. This could be attributable to active phenolics and flavonoids in the methanol extract, which reacts with superoxide radicals, hydroxyls, and lipid peroxy radicals and prevent the reactive oxygen species induced by *H. pylori* infection from damaging the gastric mucosa. Additionally, some purified plant components were considered as *H. pylori* urease inhibitors. Biological evaluation and molecular docking analysis of the reactions between urease and herbal bioactive constituents also supported the discovery and synthesis of novel plant-derived chemotherapeutics (Hassan and Žemlička 2016).

Recently, studies have found that combining phytomedicine with standard triple therapy has comparable eradication rates to the bismuth quadruple regimen after two weeks, whereas relief of clinical symptoms is greater than the bismuth quadruple regimen (Xinjie and Yan 2018). Zhang and Lan (2020) reported that TCM can be used in combination with bismuth quadruple therapy with the purpose of improving the eradication rates. Also, Judaki et al. (2017) demonstrated that a combination of curcumin (turmeric extract) with standard triple regimens considerably ameliorates oxidative stress*,* decreases DNA damage, improves the histopathological changes in chronic gastritis and increases the *H. pylori* eradication rates. Additionally, Kwiecien et al. revealed the antioxidant and antimicrobial properties of curcumin, which significantly reduced the *bacterial load* in the gastric epithelium and decreased the activities of lipid peroxidases and urease in infected rats. Despite the great potential of medicinal herbal extracts to assist treatment, they cannot be applied as monotherapy for *H. pylori* eradication.

### Vitamins

Vitamins, such as vitamins C and E, may possibly reduce the oxidative reactions and N-nitrosamine in the gastric lumen, eradicate active oxygen species and exert antioxidant effects, which may protect the host from gastric tumors (Hu et al. 2020). In 2019, Li et al. considered the impact of supplementation of *H. pylori* therapy with vitamins C and E and reported that vitamin supplementation could diminish the frequency and mortality rates of gastric malignancies in infected individuals*.* Besides, vitamins C and E were shown to possess significant inhibitory effects on *H. pylori* intensities and neutrophils’ activities. Vitamin C and E levels were found to decrease in *H. pylori*-infected individuals and rise soon after eradicating the infection. Yang-Ou et al. (2018) reported that the supplementation of vitamins C and E at elevated concentrations to *H. pylori* eradication regimes could enhance the efficacy through their antioxidant effects. Also, recent research has revealed an association between levels of vitamin D and *H. pylori* eradication. It was reported that the normal vitamin D levels in *H. pylori*-positive individuals were less than their levels in *H. pylori*-negative individuals, and patients with vitamin D deficiency showed lower eradication rates.

### Innovative drug targets to eradicate *H. pylori*

Various in silico studies, alongside genomic, metabolomic and proteomic research, have been employed to find potential novel drug targets, including *H. pylori* proteins whose homologous are lacking in their host and gut microbiome. Some recently identified targets have already been authenticated and examined using molecular docking and structural techniques to find novel inhibitors, which will require validation. Also, several attempts exist to develop effective drug delivery strategies (Roszczenko-Jasińska et al. 2020).

### Targeting biofilms of *H. pylori*

**Fig. 3:**
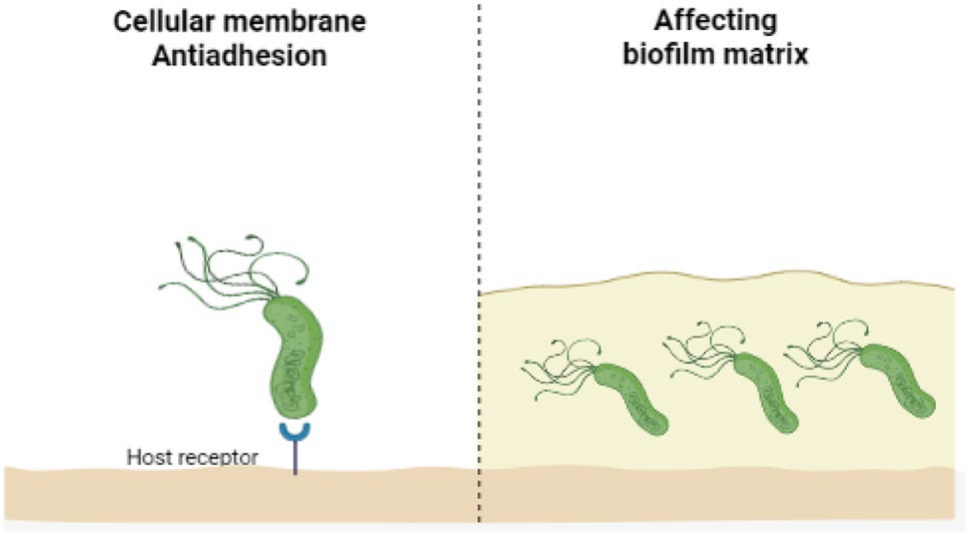
Possible targets of antibiofilm therapy

Biofilm formation is one of the challenges that face standard *H. pylori* treatments and affects their eradication efficacy thru their role in antibiotic resistance and tolerance. Despite the recent interest in *H. pylori* infection and management, biofilms are still quite understudied. Little information is available about the mechanisms involved in antibiotic tolerance and resistance (Hathroubi et al. 2020b; Zhang et al. 2023). Multiple techniques, including genomic, proteomic, transcriptomic, and target-specific investigations, have been employed to determine the factors affecting biofilm formation. However, the molecular pathways need to be clarified. Consequently, approaches that target biofilms could be useful in overcoming *H. pylori* drug resistance (Roszczenko-Jasińska et al. 2020; Abadi and Ierardi 2019) (Fig. 3).

Alternate treatment strategies which act on *H. pylori* biofilms have displayed some encouraging outcomes. Most anti-biofilm agents are generally natural products that are secondary metabolites of microorganisms, as antimicrobial peptides, biosurfactants, phytochemicals, microbial enzymes, etc. (Melander et al. 2020). Besides, a number of probiotics and quorum-sensing inhibitors have demonstrated anti-biofilm activities. This recommends the potential of some natural products to overcome *H. pylori* multidrug resistance (Hou et al. 2022). A synergistic combination of traditional antibiotics with some natural products can combat *H. pylori.* Examples include *Pistacia vera* L. oleoresin, which acts synergistically with levofloxacin to conquer antibiotic-resistant *H. pylori*. Also, *Lactobacillus salivarius* LN12 cell-free supernatant, when combined with AMX and CLR, disturbed the biofilm structures of resistant strains. Furthermore, recent studies revealed that the natural product acted synergistically with the five conventional anti-*H. pylori* antibiotic to inhibit the switch of *H. pylori* from helical to coccoid form was Myricetin (Krzyżek et al. 2021).

Recently, nanomaterials have been employed to eradicate *H. pylori* biofilms and overcome resistance (Hou et al. 2022*).* Grande et al. (2020) revealed that synthesized silver ultra-nanoclusters, when used alone or combined with metronidazole show significant anti-biofilm and antimicrobial activity. Nano-drugs, such as berberine derivatives and rhamnolipids (RHL), effectively penetrate the mucus layer and disrupt *H. pylori* biofilm (Hou et al. 2022*).*

In this regard, clinical trials revealed that N-acetylcysteine (NAC) showed activity against *H. pylori* biofilms. NAC is a thiol-containing antioxidant which disrupts mucus and is often utilized in the management of chronic respiratory tract infections. Recent research has demonstrated that NAC can inhibit the production of extracellular polysaccharide substrates, inhibit bacterial adhesion, disturb biofilms of different bacteria, and decrease the viability of sequestered cells. NAC pretreatment before initiating triple therapy improved *H. pylori* eradication rates in refractory *H. pylori* infections (Roszczenko-Jasińska et al. 2020).

Additionally, *Hathroubi* et al. (Hathroubi et al. 2020b) reported that biofilm formation in *H. pylori* results in an antibiotic tolerance state, which is reliant on extracellular proteins to some extent. The authors reported that biofilm cells are extremely tolerant to amoxicillin and tetracycline and moderately tolerant to clarithromycin. Also, they reported that the clarithromycin tolerance could be relieved by treatment with proteinase K at low levels.

### Targeting *H. pylori* virulence factors

A different approach to managing and overcoming infections is to prevent virulence instead of bacterial viability. This approach is more advantageous than the traditional antibiotic therapy. First, it does not select resistant strains and does not disrupt the gut microbiota. Second, several virulence factors are easily accessible to extra-cytoplasmic molecules. Moreover, the structure of several virulence factors has been elucidated, so novel inhibitors could be designed and investigated in silico using molecular docking techniques (Roszczenko-Jasińska et al. 2020; Sonbol et al. 2019).

*H. pylori* has a precise helical shape which improves bacterial movement thru the gastric viscous mucus layer. Numerous proteases that act on the peptide chains of peptidoglycan can have a significant role in determining cell shape. For example, Csd4 was investigated in-vitro as a novel drug target generated through structure-based analysis and investigation of the Csd4–inhibitor complex. The Csd4–inhibitor was found to alter the *H. pylori* helical form (Liu et al. 2016).

Also, T4SS and its effector molecule CagA are the main virulence factors involved in gastric carcinogenesis. Hindering the T4SS system in cagA-positive patients could be a promising treatment strategy. Hilleringmann et al. (2006) identified a small compound which blocks CagA transport. Besides, pretreatment with this compound significantly reduced *H. pylori* colonization of the mice’s gastric epithelial cells. Similarly, Shaffer et al. (2016) identified two chemical compounds which interfere with CagA and peptidoglycan translocation. Proteins of the disulphide bond (Dsb) system generate the disulphide bonds between the proteins’ cysteine residues. Accordingly, Dsb systems control protein structures and functions. *H. pylori* Dsb system isn’t the same as the Dsb systems of Gram-negative bacteria. Thus, its prospective inhibitor wouldn’t disturb the human microbiota (Bocian-Ostrzycka et al. 2015). Interestingly, quite a lot of OMPs that have a key role in *H. pylori* adherence to the host epithelial cells also have cysteine residues. The appropriate conformation of these OMPs is controlled by the activity of Dsb proteins. Also, DsbI, another element of the *H. pylori* Dsb system, hinders *H. pylori* colonization process (Godlewska et al. 2006). As a result, proteins of the *H. pylori* Dsb system could be potential targets for novel therapeutics.

## Conclusion

*H. pylori* is a common global pathogen with robust geographic variations. Diseases allied with *H. pylori* infection have a substantial burden on human health. *H. pylori* infection remains mostly without symptoms, but it can develop into several gastrointestinal ailments involving chronic gastritis, peptic, duodenal ulcers, and gastric carcinoma.

Latest studies demonstrated that the pathogenesis of *H. pylori* infection and disease outcomes is mediated by composite interactions between the host, environmental and bacterial virulence factors. Several virulence factors have a fundamental role in the pathogenesis of *H. pylori*, such as vacuolating cytotoxins (VacA), superoxidase dismutase (SOD), superoxidase dismutase (SOD), neutrophil-activating protein (NAP), catalase (KatA), cytotoxin-associated gene A product (CagA), and outer membrane porins.

Several international guidelines have recommended treating *H. pylori* infection with eradication. Suitable treatment could have promising eradication rates without recurrence of infection. Several recommended regimens exist for eradicating *H. pylori*. The standard conventional proton pump inhibitor (PPI)-based triple therapy (PPI and two antibiotics) is the most frequently utilized therapy. Also, various second-line treatments can be applied, for instance, BSQ therapy (PPI, bismuth, metronidazole, and tetracycline) is usually recommended after the failure of standard triple therapy***.*** Recently, the eradication rates of the standard *H. pylori* treatment regimens have been declining worldwide. Treatment failure is multifactorial and requires further study. Challenges that face *H. pylori* management include antibiotic resistance, reinfection, patient compliance to recommended regimens, and treatment adverse effects.

In fact, antibiotic resistance is the key challenge to the present eradicating regimens of *H. pylori*. Indeed, *H. pylori* antibiotic resistance has extended to alarming levels globally. Overuse of antibiotics, changes in the host microbiome, mutations, and incomplete treatment have resulted in alarming antibiotic resistance. Thus, there is a crucial need to find efficient alternatives to combat this global pathogen. Also, the development of *H. pylori* vaccine is a current research issue. One of the novel approaches is dual therapy with AMX and PPI. Dual therapy has proven encouraging effects in eradicating *H. pylori* and reducing the concentration of antibiotics used, thus decreasing the adverse effects on gut microbiota. Also, rifabutin-based regimens and AMPs have shown promising potential as efficient alternatives for antibiotics to combat resistant *H. pylori* infections. Moreover, the administration of probiotics in combination with the eradication regimen resulted in a greater eradication rate, fewer treatment-induced diarrhea, lower adverse effects, better immune responses and greater patient compliance. Besides, recent studies have recommended combining medicinal herbs such as turmeric extract (curcumin) with the standard triple therapy. This considerably ameliorates oxidative stress, decreases DNA damage, improves the histopathological changes in chronic gastritis and increases eradication rates.

Additionally, various in silico analyses, alongside genomic, metabolomics, and proteomic analysis, have been employed to discover potential novel drug targets, including *H. pylori* proteins whose homologs are lacking in their host and gut microbiome. Some recently identified targets have already been validated and investigated by molecular docking and structural analysis to find novel inhibitors, which will also require validation. Besides, there are several attempts to develop effective drug delivery strategies. Approaches targeting biofilms could be useful in overcoming *H. pylori* antibiotic resistance. Also, *H. pylori* proteins of the Dsb system could be possible targets for novel therapeutics. Novel anti-*H. pylori* treatment strategies and *H. pylori* databases globally are warranted in the near future.

## Data Availability

All data are available in the manuscript.
